# Microcontact Peeling as a New Method for Cell Micropatterning

**DOI:** 10.1371/journal.pone.0102735

**Published:** 2014-07-25

**Authors:** Sho Yokoyama, Tsubasa S. Matsui, Shinji Deguchi

**Affiliations:** 1 Department of Nanopharmaceutical Sciences, Nagoya Institute of Technology, Nagoya, Japan; 2 Department of Biomolecular Sciences, Tohoku University, Sendai, Japan; University of California, San Diego, United States of America

## Abstract

Micropatterning is becoming a powerful tool for studying morphogenetic and differentiation processes of cells. Here we describe a new micropatterning technique, which we refer to as microcontact peeling. Polydimethylsiloxane (PDMS) substrates were treated with oxygen plasma, and the resulting hydrophilic layer of the surface was locally peeled off through direct contact with a peeling stamp made of aluminum, copper, or silicon. A hydrophobic layer of PDMS could be selectively exposed only at the places of the physical contact as revealed by water contact angle measurements and angle-resolved X-ray photoelectron spectroscopy, which thus enabled successful micropatterning of cells with micro-featured peeling stamps. This new micropatterning technique needs no procedure for directly adsorbing proteins to bare PDMS in contrast to conventional techniques using a microcontact printing stamp. Given the several unique characteristics, the present technique based on the peel-off of inorganic materials may become a useful option for performing cell micropatterning.

## Introduction

Cell micropatterning is a technique for spatial control of the adhesive regions for individual cells and/or cell colonies. This artificial control enables highly reproducible experiments and has provided many interesting findings in cell biology [1]. Among them include the force-dependent mechanisms of the spindle orientation determined by the geometry of the extracellular matrix [2], [3], the subcellular localizations of focal adhesions determined by the distribution of traction stresses [4]–[6], and the induction of apoptosis [7], [8] or differentiation [9], [10] via controlling the size/shape of micropatterns. Thus, micropatterning is becoming a powerful tool particularly in mechanobiology studies [1] as well as in tissue engineering, cell-based biosensors, biological assays, and drug screening [11]–[13].

Among numerous micropatterning techniques, microcontact printing (µCPr) has become one of the most popular [1], [12]. For µCPr, a polydimethylsiloxane (PDMS) stamp with desired micro-features is used to print extracellular matrix (ECM) proteins onto particular areas of cell culture substrates [13], [14]. The remaining regions are blocked with protein-resistant chemicals such as Pluronic F-127 or bovine serum albumin. Cell adhesive regions can thus be selectively produced, thereby allowing for control of cell morphology and localizations. However, the effects of µCPr on the conformation of proteins to be transferred are not fully understood [12]. Indeed, some proteins have been reported to be disrupted in structure upon adsorption to bare PDMS [15]–[17]. Thus, technical alternatives that avoid the protein adsorption are expected to appear to diversify the approach to micropatterning for a variety of proteins.

Here we describe a new technique for cell micropatterning that does not comprise a procedure for the adsorption of target biomolecules to bare PDMS. We show that an oxygen-rich surface layer grown on PDMS substrates is physically peeled off in a spatially selective manner with direct contact to a stamp having a relatively high surface energy compared to PDMS. Water contact angle measurements and X-ray photoelectron spectroscopy (XPS) suggested that the oxygen layer was transferred to the peeling stamp, allowing for selective adhesions of ECM and cells to the unpeeled hydrophilic regions. We discuss the potential of this new technique, which we call microcontact peeling (µCPe), to become a future practical option for cell micropatterning.

## Materials and methods

### Cell culture

U2OS cells (a human osteosarcoma cell line; ATCC) were cultured in high-glucose (4.5 g/L) DMEM (Invitrogen) with 10% fetal bovine serum (SAFC Biosciences) and 1% penicillin–streptomycin (Invitrogen) in a 5% CO_2_ incubator at 37°C.

### Peel-off of inorganic layers

**Figure 1:**
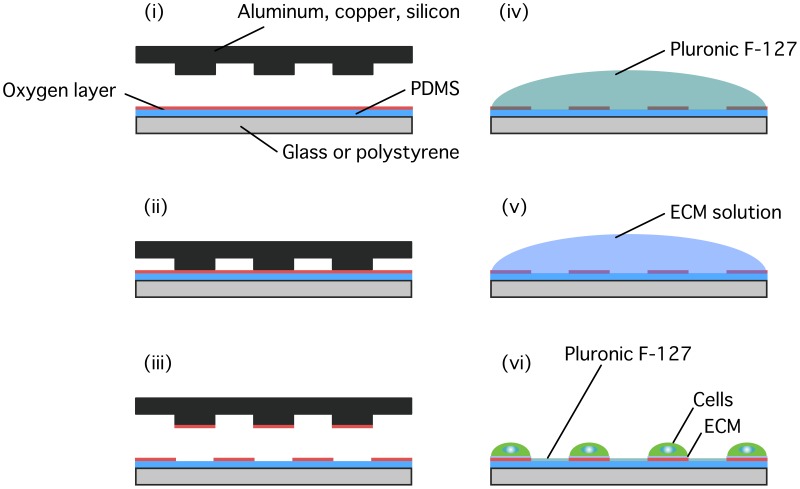
Schematic of µCPe. Glass or polystyrene dishes are coated with PDMS and then treated with oxygen plasma to form an oxygen-rich layer at the surface (i). A stamp made of aluminum, copper, or silicon was brought into physical contact with the substrates (ii), and removed away to peel off the oxygen-rich layer at the places of the contact (iii). The substrates were then treated with Pluronic F-127 (iv) and ECM (in the present study, gelatin) (v). ECM and cells adhere selectively to the unpeeled regions (vi).

The surface of 35-mm glass-bottom dishes or polystyrene dishes was coated with polydimethylsiloxane (PDMS; prepared at w/w ratios of 10–50 for the base polymer to 1 for the cross-linker; Sylgard 184, Dow Corning) using a spin-coater, degassed, and oven cured at 60°C for 10 h. The PDMS surface was exposed to oxygen plasma (4 mA, 20 Pa) for 1 min using a plasma generator (SEDE-P, Meiwafosis). A copper electron microscopy (EM) grid (G203, EM Japan), copper sheet (Taiho, Eggs), aluminum sheet (Toyo Aluminium Ekco Products), or silicon wafer (Kyodo International) was put on the plasma-treated surface of PDMS (Fig. 1), and the dish was laid sideways and centrifuged using a spin-coater (K-359S1, Kyowariken) at ∼1,500 rpm. The upper material, which presses a part of the PDMS during the centrifugation and thus functions as a stamp, was removed away using forceps to peel off the surface layer of PDMS. In a separate experiment, a peeling stamp was pressed manually by hand to the plasma-treated substrate instead of centrifugation. The surface of PDMS was then treated with 0.2% Pluronic F-127 (Invitrogen) for 1 h and coated with 0.1% gelatin (Sigma) in PBS for 4 h at 37°C for subsequent cell culture.

### Fluorescence microscopy

Cells were cultured on the pretreated dish overnight, fixed with 4% paraformaldehyde for 15 min, and permeabilized with 0.1% Triton X-100 for 5 min. F-actin and nuclei were fluorescently labeled with Alexa 488-conjugated phalloidin (Molecular Probes) and Hoechst 33342 (Molecular Probes), respectively. In a separate experiment, Alexa 488-conjugated fibrinogen (Molecular Probes) was coated on the substrate prior to cell seeding. Images were taken using a camera (ORCA-R2, Hamamatsu) under a microscope (IX-71, Olympus).

### Angle-resolved XPS

Changes in surface composition during the peel-off process were investigated using angle-resolved XPS (PHI 5000 VersaProbe II; Ulvac-Phi). Monochromated aluminum *Kα* 945 radiation (25 W, 15 kV) was employed with an angle of 10° between the sample surface and the entrance to the electron analyzer, which produced a predominant signal orienting from the top surface. Survey spectra were acquired in a binding energy range of 0–1,200 eV. High-resolution spectra of C1s, O1s, Al2p, and Si2p were then collected, and integrals of each of the intensities were calculated in software (Common Data Processing System ver. 11). The ratio of C, O, Al, or Si to the total was obtained to quantify the elemental composition of the surface layer. Statistical differences were analyzed using the unpaired Student's *t*-test, with a significance level of *p*<0.01.

### Measurement of contact angles

A water droplet of 4 µl was placed using a micropipette on the surface of PDMS, oxygen plasma-treated PDMS, copper sheet, aluminum sheet, or silicon wafer. Contact angles between the water droplets and the surfaces were observed with a 90°-tilted stereomicroscope (S8APO, Leica) and imaged with a camera (Moticam 1000, Motic). Images were analyzed using ImageJ (NIH) to obtain the contact angles. The measurements were performed at various points over the entire surface of the materials. The surface was evaluated to be hydrophilic and hydrophobic when the water contact angle was <90° and ≧90°, respectively, by reference to Förch et al. [18].

### Reusability of identical silicon wafers

Repeatability of the peel-off effect using identical silicon wafers was investigated. A silicon wafer was first used as a peeling stamp for plasma-treated PDMS, and the effect of the peel-off on cell adhesions was observed. The silicon wafer was then subjected to ultrasonic cleaning (40 kHz, 70 W; VS-70RS1, Velvo-Clear) for 20–40 min, and again used as a peeling stamp for another plasma-treated PDMS to confirm whether the cleaned silicon wafer still possessed the ability of peeling off the oxygen surface layer and allowed for selective cell adhesions. For each step, water contact angles were measured on the identical silicon wafers to evaluate changes in hydrophilicity.

## Results

### Spatially selective separation of functionalized surface layer of PDMS

**Figure 2:**
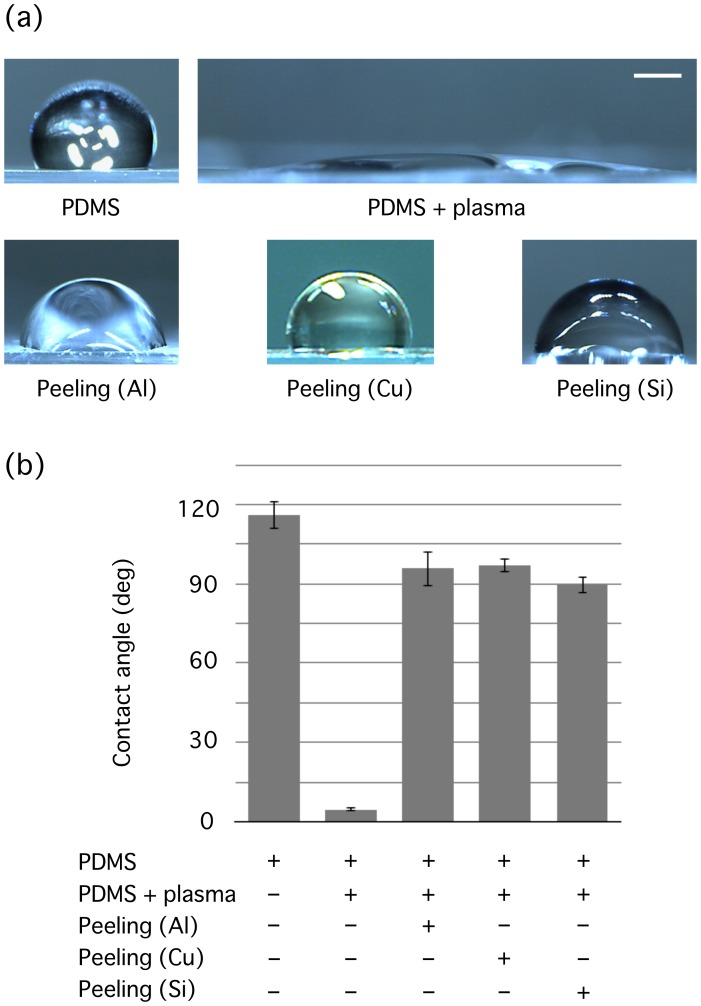
Contact angles between the substrates and ultrapure water. (a) Side views of a water droplet on bare PDMS, oxygen plasma-treated PDMS (PDMS + plasma), oxygen plasma-treated PDMS made contacted with an aluminum sheet (Peeling (Al)), oxygen plasma-treated PDMS made contacted with a copper sheet (Peeling (Cu)), or oxygen plasma-treated PDMS made contacted with a silicon wafer (Peeling (Si)). Representative data from *n* ≧ 3. Scale bar, 1 mm. (b) Quantified contact angles at each condition. Data are represented by mean ± SD.

The purpose here is to demonstrate that an oxygen-rich layer grown on a PDMS surface is transferred onto an aluminum material through direct physical contact, which thus allows for local modification of the surface property. The change in the surface wettability during the experimental process was quantified by measuring the contact angle for ultrapure water, which is negatively correlated with hydrophilicity [18]. The contact angle was initially 116 ± 5° (mean ± SD) for the untreated PDMS surface being hydrophobic, while dropped sharply to 5 ± 1° upon the oxygen plasma treatment to become hydrophilic (Fig. 2). An aluminum sheet was placed in conformal contact with and then removed away from the highly hydrophilized surface, which resulted in an increase in contact angle at the PDMS surface to 96 ± 7° and thus returned to a hydrophobic state.

**Figure 3:**
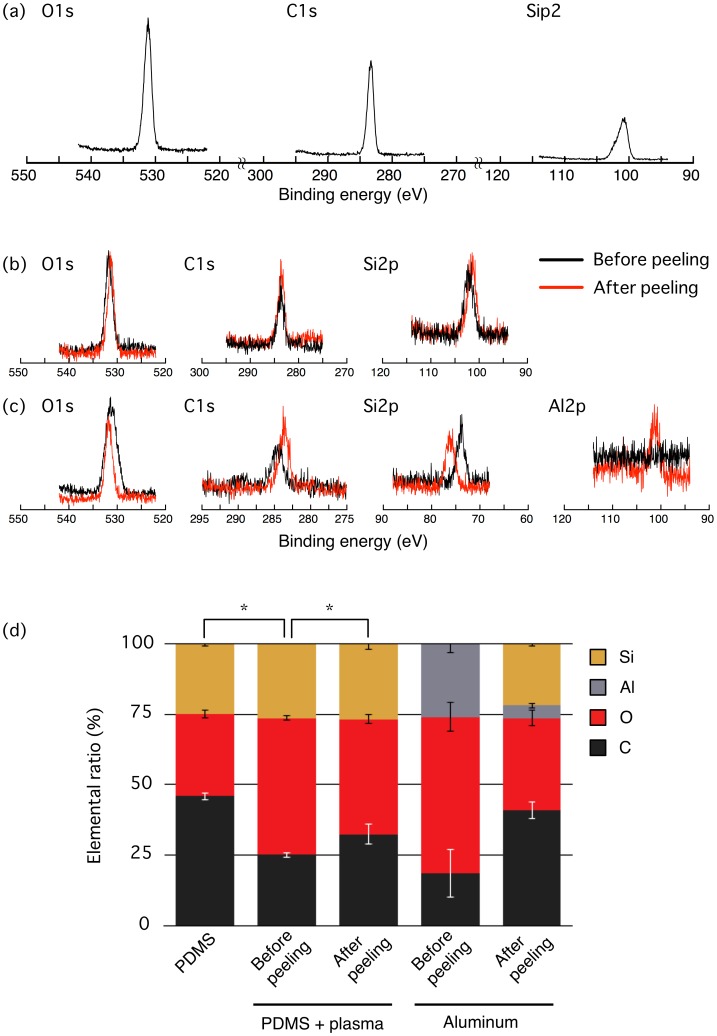
Transfer of the surface layer of PDMS onto an aluminum peeling stamp investigated by angle-resolved XPS. (a) C1s, O1s, and Si2p spectra of an untreated PDMS. Representative data from *n* = 3. (b) High-resolution spectra of C1s, O1s, and Si2p obtained at the surface of oxygen plasma-treated PDMS before and after peel-off with the aluminum sheet. Representative pair from *n* = 3. (c) High-resolution spectra of C1s, O1s, Al2p, and Si2p obtained at the surface of the aluminum sheet before and after peel-off. Representative pair from *n* = 2. (d) Quantified changes in the elemental ratio. Data are represented by mean ± SD. The asterisks represent a significant difference in the compositional ratio of oxygen measured for *n* = 3 separate measurements.

These macroscopic observations on wettability were corroborated by angle-resolved XPS in which the elemental ratio at the surfaces was measured (Fig. 3). Three major peaks of C1s, O1s, and Si2p were detected in survey scan and were investigated at high-resolution to examine the major components of PDMS, i.e., carbon, oxygen, and silicon (Fig. 3a and 3b). For aluminum sheets, another peak of Al2p was detected in survey scan and was also analyzed at high-resolution (Fig. 3c). The integral of the intensities obtained was calculated at each of the peaks to quantify the compositional ratios. The content percentage of oxygen at the surface of PDMS significantly increased upon the oxygen plasma treatment from 30.73% (PDMS, Fig. 3d) to 48.35% (Before peeling, PDMS + plasma; Fig. 3d), while decreased after the physical contact with the aluminum sheet to 41.52% (After peeling, PDMS + plasma; Fig. 3d). In contrast, silicon was detected at the surface of the aluminum sheet only after the physical contact with the plasma-treated PDMS to have a ratio of 21.84% (After peeling, Aluminum; Fig. 3d). The ratio of oxygen on the surface of the aluminum sheet was reduced on average after the peel-off probably because the aluminum sheet was originally oxidized to be Al_2_O_3_; but still, the absence and appearance of Si before and after the peel-off, respectively, as well as the sharp decrease in the ratio of Al (Fig. 3d) indicate that the surface layer of PDMS was peeled off and transferred onto the aluminum sheet.

### Selective adhesion of cells to unpeeled region

**Figure 4:**
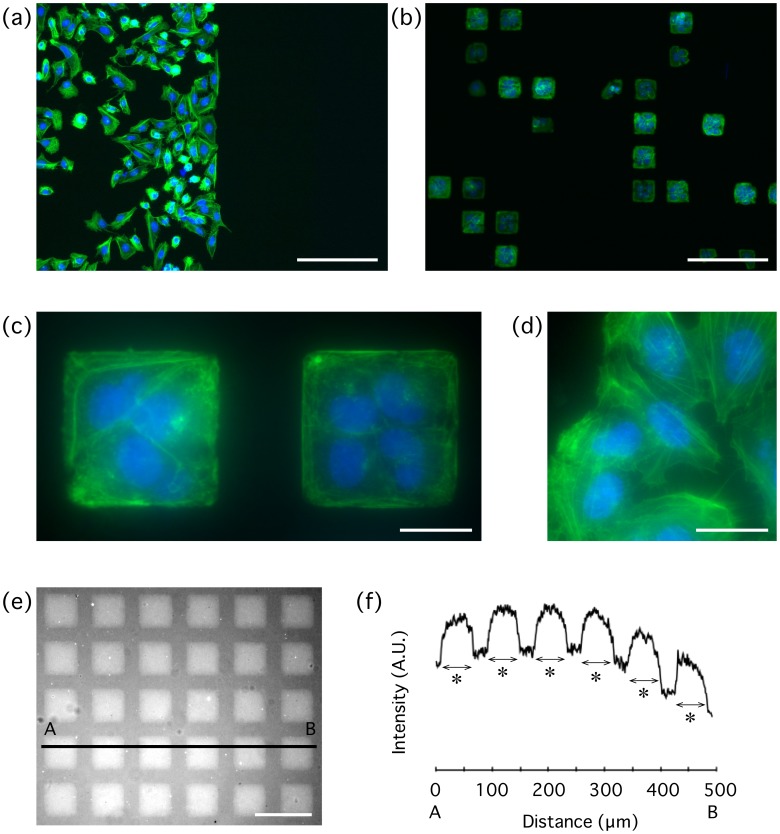
Representative images of spatially selective cell adhesions by µCPe. (a) Cells adhere to unpeeled regions (left) but not to peeled regions (right) created by physical contact with an aluminum sheet. (b) Demonstration of the selective cell adhesion at a micro-scale using a copper EM grid with a square mesh length of 54 µm. Cells are restricted to spread within square islands. (c) A high magnification view of the cells on the square micropatterns. (d) Cells outside the micropatterns (allowed to spread freely) observed with the same magnification as that of c. (e) Coating with fluorescent fibrinogen visualizes square adhesive regions. (f) Intensity profile along the line A–B in e. Asterisks represent 54 µm. F-actin and nuclei are shown in green and blue, respectively. Scale bars, 200 µm (a and b); 25 µm (c and d); 200 µm (e).

We seeded cells onto the PDMS substrates that experienced the successive treatments with oxygen plasma, physical contact with the aluminum sheet, Pluronic F-127, and finally gelatin (Fig. 4a). The treatment with Pluronic F-127 is known to hydrophilize an originally hydrophobic surface while preventing the binding of proteins. The results showed that cells were adhered selectively to the surface that did not experience the physical contact with the aluminum sheet, and were spread and grown normally on the surface (Fig. 4a, left). Meanwhile, cells were totally absent on the other areas that were previously subjected to the physical contact (Fig. 4a, right). Thus, cell adhesive regions could selectively be provided with the physical peeling-off of the oxygen layer.

### Selective peeling-off enables micropatterning of cells

To next demonstrate, at a micro-scale, the effectiveness of the peel-off on selective cell adhesions, we used a copper EM grid as a peeling stamp. The EM grid has square-mesh features with an inner edge length of 54 µm. The EM grid was uniformly pressed onto the surface of plasma-treated PDMS and then removed away aiming at peeling off the oxygen-rich layer as successfully achieved at a macroscopic scale by the aluminum sheet (Fig. 4a). The results showed that cells were selectively adhered to unpeeled areas, and individual cells were confined to narrow square areas with the same size as that of the grid meshes (Figs. 4b, 4c, and 4d). In a separate sample, we treated the physically manipulated PDMS with fluorescent fibrinogen to distinguish between ECM-philic and ECM-phobic regions (Fig. 4e). Fluorescence intensity was low outside the 54×54-µm^2^ square regions (Fig. 4f), indicating that the contacted places were modified to be ECM-phobic.

We also measured the water contact angle to evaluate the efficiency of the peeling-off with copper (Fig. 2). A copper sheet was pressed onto the plasma-treated PDMS and then removed away. The contact angle of the plasma-treated PDMS increased to 97 ± 3° for peeled conditions (Peeling (Cu), Fig. 2), suggesting that the oxygen surface layer of PDMS is effectively transferred to copper as well.

### Silicon peeling stamps are reusable

**Figure 5:**
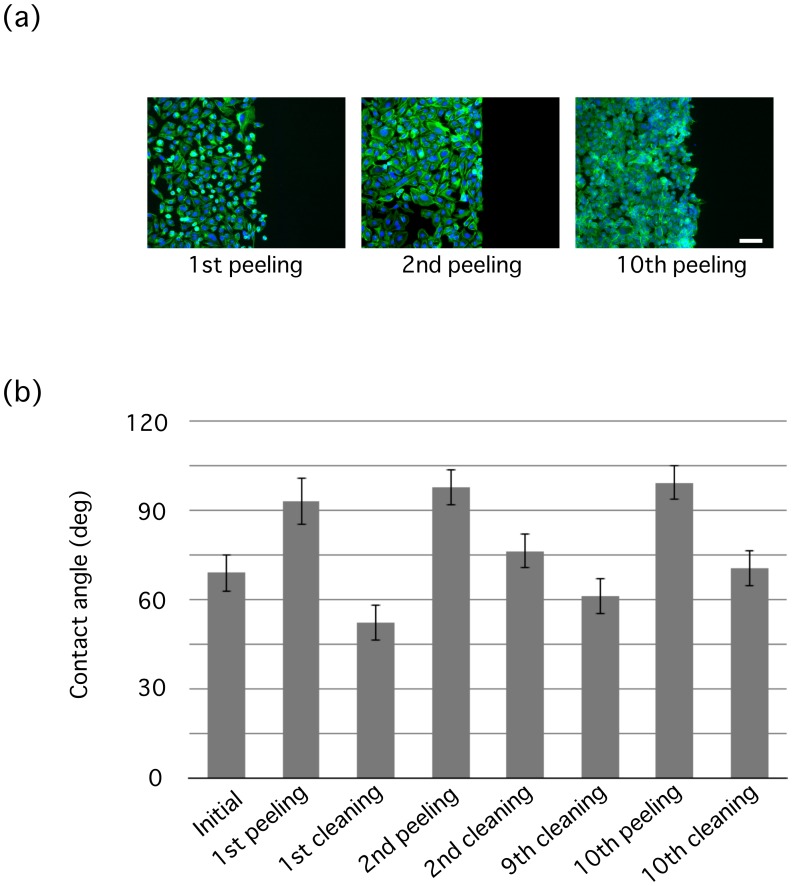
Repeatable use with identical silicon wafers. (a) Cells adhere to unpeeled regions (left) but not to peeled regions (right) created by physical contact with a silicon wafer. 1st, 2nd, and 10th peelings represent selective cell adhesions using the same silicon stamp without ultrasonic cleaning, after 1st cleaning, and after 9th cleaning, respectively. F-actin and nuclei are shown in green and blue, respectively. Scale bar, 100 µm. (b) Contact angles for the same silicon wafers measured in sequence before the first peeling-off (Initial), after the first peeling-off (1st peeling), after the subsequent cleaning (1st cleaning), after the subsequent second peeling-off (2nd peeling), after the ninth cleaning (9th cleaning), after the tenth peeling-off (10th peeling), and after the tenth cleaning (10th cleaning). Data are represented by mean ± SD (*n* = 3).

We tested the usefulness of a silicon wafer as a peeling stamp. A planar silicon wafer was pressed onto the plasma-treated PDMS, and cells were seeded on the surface after the removal of the wafer and the subsequent treatments with Pluronic F-127 and gelatin. We observed spatially selective adhesions of cells to unpeeled areas (1st peeling, Fig. 5a). Contact angle measurements showed an increase from 5 ± 1° for unpeeled plasma-treated PDMS (PDMS + plasma, Fig. 2b) to 90 ± 3° for peeled conditions (Peeling (Si), Fig. 2b) to become hydrophobic, supporting that the oxygen layer created on PDMS with plasma treatment is effectively transferred to the silicon materials.

For future practical use, it is expected that micro-featured silicon wafers can be repeatedly used for µCPe; otherwise, the wafer has to be expendable regardless of effort and expense for its production. We treated the silicon wafers with ultrasonic cleaning after using them as a peeling stamp, and tested whether they retrieved the ability of peeling off oxygen layers from plasma-treated PDMS. Silicon wafers had a contact angle of 69 ± 6° initially before use (Initial, Fig. 5b) and 93 ± 8° after use as a peeling stamp (1st peeling, Fig. 5b). The ultrasonic cleaned silicon wafers exhibited a low contact angle of 52 ± 6° (1st cleaning, Fig. 5b). Using these re-hydrophilized wafers as a peeling stamp, selective cell adhesions were achieved again (2nd peeling, Fig. 5a), which resulted in an increase in the contact angle of the silicon wafers to 98 ± 6° (2nd peeling, Fig. 5b). We further observed similar selected cell adhesions using identical silicon stamps that experienced the peel-off/cleaning set 10 times (10th peeling, Fig. 5a). Contact angles of the silicon surface consistently decreased (61 ± 3°) and increased (99 ± 3°) to become hydrophilic and hydrophobic after the 9th ultrasonic cleaning and 10th peel-off, respectively (9th cleaning, 10th peeling, Fig. 5b). Finally, another ultrasonic cleaning returned the silicon wafer to a hydrophilic state with a contact angle of 71 ± 4° (10th cleaning, Fig. 5b). We thus confirmed the reusability of the silicon wafers as a peeling stamp even after 10-times use.

## Discussion

We described a new cell micropatterning technique using a stamp to physically peel off the surface oxygen-rich layer of plasma-treated PDMS. We called this technique µCPe after µCPr, the latter of which is a widely used micropatterning technique and employs a PDMS stamp to print ECM onto a glass or polystyrene surface. For µCPe, we showed that at least aluminum, copper, and silicon are available as a stamp to peel off the surface layer present on PDMS.

The content ratio of oxygen increased at the surface of PDMS upon the exposure to oxygen plasma (Before peeling, PDMS + plasma; Fig. 3d), and then hydrophilicity was induced as quantified by the decrease in water contact angle (PDMS + plasma; Fig. 2b). This hydrophilization is consistent with previous reports that submitted a surface modification of PDMS upon oxygen plasma treatment from Si-CH_3_ to Si-COOH, Si-OH, or Si_2_-O within the repeated molecular architecture of dimethylsiloxane Si(CH_3_)_2_-O- that PDMS initially has [19]–[21]. The decrease in oxygen ratio after the physical contact (After peeling, PDMS + plasma; Fig. 3d) and the resulting recovery to a hydrophobic state (Peeling (Al); Fig. 2b) suggest that the surface layer containing enriched oxygen is peeled off, and unmodified PDMS is exposed again. Aluminum, copper, and silicon should all be higher than PDMS in surface energy because surface energy basically correlates with water contact angle in a positive manner [12], [18]. Thus, the surface oxygen layer is likely to be transferred onto the physically touched materials, and consequently cells adhere selectively to the unpeeled areas of PDMS via the remaining oxygen layer as cells are normally grown on these hydrophilized regions [22]. This enables patterning of cells even at a micro-scale if micro-fabricated stamps (including EM grids as demonstrated in the present study) are employed (Fig. 4) because the transfer of oxygen layers occurs only at places of the physical contact. For the molecules to be separated from PDMS, the binding to the new surface must be more energetically favorable than staying on PDMS, while the low surface energy of PDMS would allow it to easily occur.

The new technique µCPe may be apparently similar to well-established µCPr as they both take advantage of the difference in inherent surface energy between PDMS and a material to which molecules are transferred. Here we discuss the potential of µCPe while focusing on the difference in technical procedure between µCPe and µCPr. Biomolecules to be transferred are directly adsorbed to bare PDMS in both traditional [12] and recently modified µCPr [23]–[26], the latter of which utilizes a peel-off of ECM proteins from the PDMS. Some proteins were altered in the native structure upon adsorption to PDMS [15]–[17]. Thus, the effects of the adsorption to bare PDMS on the conformation of proteins are in general not fully understood [12]. In the case of µCPe, the molecules transferred through physical contact are neither proteins nor cells but are inorganic oxygen layers having simpler molecular structures. Thus, micropatterns with less denatured target proteins might be efficiently obtained in µCPe; further research is needed to test this hypothesis.

For traditional µCPr, micro-featured PDMS stamps are more or less deformed during printing by applying vertical pressure such that the stamps collapse or buckle against the stiff substrates, consequently resulting in ill-defined patterns [11], [12], [27]. These issues were greatly improved by employing a planar PDMS stamp that is subjected to successive stamping procedures onto firstly a fine-featured template and secondly a flat substrate [23]–[25]. In these modified µCPr using a planar PDMS, the deformation of the stiff template is ignorable during the application of vertical pressure, thereby allowing for accurate micropatterning with sub-100-nm resolution. Likewise, µCPe employs a planar PDMS, and the deformation of stiff peeling stamps (made of aluminum, copper, or silicon in the case of the present study) must be ignorable. Evaluation of the spatial resolution achieved by µCPe through more thorough preparation of a fine-featured peeling stamp will be the subject of future investigation.

The liquid-free procedure for µCPe leads to concise experiments. Specifically, the absence of an experimental procedure for immersing PDMS stamps into liquids for handling ECM lessens the chance of contamination, and thus aseptic working conditions are stably preserved in µCPe. Importantly, the dry dish allows for easy centrifugation together with a peeling stamp and consequently for homogeneous vertical pressure onto the planar PDMS, which, according to our preliminary experiments, dramatically stabilized the spatial accuracy of the patterning. On the other hand, manual stamping sometimes resulted in uneven printing due to unavoidably inhomogeneous application of the vertical pressure.

We examined the utility of µCPe at a micro-scale using EM copper grids (Fig. 4). We also observed that silicon wafers are also useful as a peeling stamp (Fig. 5). In addition, the oxygen layer transferred to the silicon can be removed away simply by ultrasonic cleaning, suggesting that one silicon stamp can produce multiple copies of a pattern. Thus, for future applications, micro-fabricated silicon wafers designed by individual researchers for their aims may be used as a peeling stamp to obtain arbitrary micropatterns.

In summary, we developed µCPe, a new cell micropatterning technique. Transfer of an oxygen layer grown on PDMS substrates onto a peeling stamp and resultant surface modification into hydrophobicity were observed by angle-resolved XPS as well as contact angle measurements. The validity of µCPe at a micro-scale was provided using micro-featured stamps. Furthermore, the reusability of identical silicon wafers as a peeling stamp was demonstrated with ultrasonic cleaning. µCPe has several unique characteristics, and thus this new technique can diversify practical options for performing cell micropatterning.
